# The Use of Virtual and Immersive Technology in Creating Personalized Multisensory Spaces for People Living With Dementia (SENSE-GARDEN): Protocol for a Multisite Before-After Trial

**DOI:** 10.2196/14096

**Published:** 2019-08-19

**Authors:** Gemma Goodall, Ileana Ciobanu, Kristin Taraldsen, Jon Sørgaard, Andreea Marin, Rozeta Draghici, Mihai-Viorel Zamfir, Mihai Berteanu, Walter Maetzler, J Artur Serrano

**Affiliations:** 1 Department of Neuromedicine and Movement Science Norwegian University of Science and Technology Trondheim Norway; 2 University of Medicine and Pharmacy Carol Davlia Bucharest Romania; 3 ELIAS University Hospital Bucharest Romania; 4 Department of Neurology Christian-Albrechts-University Kiel Germany; 5 Norwegian Centre for eHealth Research University Hospital of North Norway Tromsø Norway

**Keywords:** dementia, emotions, technology, multimedia, eHealth

## Abstract

**Background:**

The number of people living with dementia is rapidly increasing. With dementia’s impact on memory, communication, and self-identity, it is important to identify ways of meeting individual needs of diagnosed individuals and their caregivers. This study will test a new intervention, SENSE-GARDEN, that integrates autobiographical music, films, pictures, and scents with innovative technology to create an immersive environment tailored specifically for the individual.

**Objective:**

The SENSE-GARDEN study is an Active Assisted Living Program–funded multicenter project. The primary objective of the study is to assess whether a personalized, innovative technology-based intervention can improve the well-being of older adults living with moderate to severe dementia. The study will also assess whether the intervention can improve coping and reduce burden in caregivers.

**Methods:**

A controlled before-after study design will be used. There will be 3 sites in 3 trial countries: Belgium, Norway, and Portugal. A total of 55 people with dementia (PWDs) will be recruited. All eligible participants for the study will be randomized into the intervention or control group. For the first three months of the study, all participants will receive the SENSE-GARDEN intervention. For the final month of the study, the intervention group will continue visits to the SENSE-GARDEN, and the control group will discontinue visits. A mixed-methods approach will be used, including the use of standardized outcome measures, quantitative physiological data, and qualitative interview data.

**Results:**

The trials commenced recruitment in August 2019, and all data are expected to be collected by the end of May 2020. A user-centered design process is underway, with results from the first phase of user interviews indicating that people with mild cognitive impairment, family caregivers, and professional caregivers consider the SENSE-GARDEN to be a potentially valuable tool in providing numerous benefits to dementia care. Feasibility testing of the SENSE-GARDEN has been completed and results are expected to be published in October 2019.

**Conclusions:**

Findings from the SENSE-GARDEN trials will provide insights into the use of technology for personalizing interventions to the PWD. This will have potential implications on not only dementia research, but it may also have influences on care practice.

**International Registered Report Identifier (IRRID):**

DERR1-10.2196/14096

## Introduction

### Background

Dementia is an umbrella term for a variety of neurodegenerative diseases that most often affect memory, behavior, and communicative abilities [1]. There are approximately 47 million people living with dementia worldwide [2]. With this number set to increase to 131.5 million by 2050, it is of the utmost importance to tackle dementia’s progressive impact on the well-being of people living with a diagnosis. The World Health Organization (WHO) has called for action on dementia, presenting it as a public health priority at a global level [1]. This action includes a call for research to identify ways of supporting the needs of people living with dementia, their caregivers, and society in the context of costs, understanding, and awareness.

People with dementia (PWDs) progressively disconnect from the world; they experience loss of function, especially memory, affecting their cognition, physical activity, and verbal and nonverbal communication. A person’s ability to communicate with others progressively worsens during the course of dementia, which can lead to the individual engaging in problem behaviors as an expression of unmet needs [3,4]. This can then result in an increase in caregiver burden for family members and residential care staff.

Continuing social contact with others and participating in activities is important for maintaining quality of life. Participating in past pleasant activities has an impact on functional ability and psychological well-being for PWDs living in residential care [5]. However, care facilities often struggle to fulfil this need for engagement and active participation, especially for residents in more advanced stages of cognitive and physical impairment [6]. This lack of external stimuli in the care environment can cause PWDs to become increasingly depressed [7].

In recent years, studies have identified numerous complex needs of PWDs living in long-term care. These include management of challenging behaviors, maintenance of social relationships, involvement of people with cognitive deficits in meaningful activities, and supporting the emotional needs of all [8,9]. Emotion-oriented approaches to care have been shown to be cost-effective ways of improving psychological well-being and social behavior among PWDs [10,11]. These nonpharmacological approaches are often person-centered, focusing on the personal, social, and emotional needs of the individual. Reminiscence rooms, virtual gardens, and virtual reality forests are examples of how immersive technologies have been integrated in emotion-oriented approaches designed to create effective interventions for PWDs [12,13]. However, this area of study has called for further research in determining what works best for the individual [6]. It has recently been suggested that an individualized multisensory environment for PWDs would be a highly beneficial intervention, especially if family members are included in the selection of stimuli [14]. Our research builds on this suggestion, creating not only a personalized multisensory intervention but one that also incorporates immersive technology, all with the inclusion of family members, friends, and professional care staff.

### SENSE-GARDEN Intervention

The study is performed as part of the SENSE-GARDEN EU project, funded by the Active Assisted Living (AAL) Program Call 2016. It is a 3-year project that brings together a consortium of partners across Belgium, Norway, Portugal, and Romania. This multidisciplinary project is embracing a user-centered design approach throughout the development and implementation of the intervention. The SENSE-GARDEN intervention addresses the need for individualized approaches to dementia care by creating a multisensory environment that automatically adapts to the individual with dementia. Through integrating autobiographical music, films, images, and scent with technology, the SENSE-GARDEN is able to offer an immersive experience tailored specifically for the individual based on an individual profile including preferences in music, images, videos, and personal media, such as family photos.

**Figure 1:**
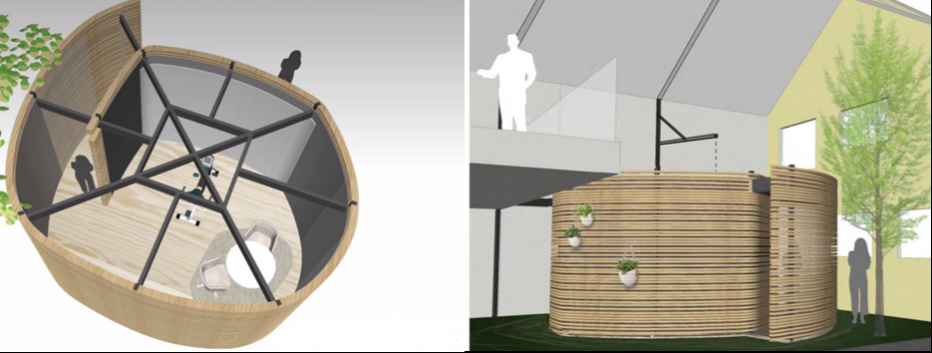
Architectural sketches of the SENSE-GARDEN space (left:interior, right: exterior).

The project aims at creating virtual spaces that are automatically adaptable to the personal memories and individual preferences of the users. The design of the space is shown in Figure 1. These spaces will be designed to strengthen the awareness of older PWDs by combining multisensory stimulation with physical activity and techniques from reminiscence therapy and Montessori methods. This stimulation of sight, touch, hearing, balance, and smell is expected to lead to a reconnection with reality for the PWD, resulting in an improvement in overall well-being and quality of life. The intervention will use a combination of various activities and approaches:

- *Reality Wall:* This is the projection of landscape videos, with some including familiar scenery and known places, onto a large wall.
- *Move to Improve:* This is an augmented reality game aimed at improving balance and increasing levels of physical activity.
- *Memory Lane:* This is an interactive touchscreen device showing family photographs and media from the individual’s life story.
- *Life Road:* This is a stationary bicycle placed in front of a film showing a familiar place to the PWD.
- *Sounds Surround Me:* These are sounds from the surrounding sound speakers playing familiar music and background soundscapes.
- *Scents to Memories:* This is an olfactory dispensary system releasing familiar scents.
- *Films of My Life:* This is a collection of classic film excerpts meaningful for the PWD, together with family movies.

The rationale to include these elements in the SENSE-GARDEN intervention stems from the current evidence base of ways to improve the well-being of PWDs. For instance, there has been an increasing amount of research on combining biographical information with multimedia apps to create digital *life stories*. Research on multimedia biography apps for PWDs has shown numerous benefits, such as stimulating reminiscence, evoking positive emotions, stimulating social interaction with others, and improving autobiographical memory [15-17].

Research also suggests that there is potential to use technology to create immersive environments in dementia care. A recent study investigated the effects of a *virtual reality forest* on PWDs, in which large screen projection of scenery such as a forest or river was used in combination with movement sensors to create an immersive and interactive environment [13]. Although this environment was not based on the biographical information of the user, results showed improved levels of pleasure and alertness during the intervention.

Considering the promising results of these studies, the innovative approach of SENSE-GARDEN has the potential to provide numerous benefits to its users. Together, it was anticipated that the integration of these components and various stimuli would complement each other to create an immersive environment that the PWD can connect with, for example, pictures of forest scenery could be combined with the sound of singing birds and the odor of a pine forest. Other scenarios may include seas, streets, the beach, urban, or rural areas according to the most meaningful memories of each individual PWD. Having this immersive environment tailored to the individual memories of each user will create a connection to the more active areas of the memory; such stimuli may result in various types of emotional states.

Another important aspect of SENSE-GARDEN is the aim to support PWDs in sharing their life story with a caregiver. The theoretical underpinnings of this aim can be linked to previous study conducted on dementia and narrative. The literature has commented on the important role of digital media in conveying narratives and supporting meaningful conversations for PWDs and their caregivers [18]. By interacting with the various stimuli together with a caregiver, it is hoped that the PWD is able to reminisce on their past and become engaged in the *present moment*. The SENSE-GARDEN encourages PWDs to exercise at both mental and physical levels and takes them back into places they feel connected to. They can, for example, cycle or walk in a well-known space and feel like they are going home. Such experiences may have an effect on invigorating their identity and helping to recover their sense of self.

Relatives will have a key role in the initial adaptation of the space by providing information regarding the past of the user. After this initial setting, the SENSE-GARDEN will automatically adjust to the individual person. Feedback during the sessions will allow the SENSE-GARDEN system to learn the preferences of each user, meaning that the sessions will become increasingly personalized with each visit.

### Objectives

The study will test operational SENSE-GARDENs installed in the 3 study sites: Belgium, Norway, and Portugal. With these countries, a large coverage of the European context is achieved, allowing to study cultural, social, economic, and legal differences between the countries and European regions. The primary objective of this study is to determine whether the delivery of the innovative technology developed in SENSE-GARDEN can improve well-being in older adults with intermediate to advanced dementia. The WHO defines health as the “state of complete physical, mental, and social well-being and not merely the absence of disease or infirmity” [19]. This study will adopt this holistic approach to health and well-being by measuring well-being through primary outcomes related to behavior, activities of daily living, cognitive function, quality of life, self-identity, communication, and social presence.

### Primary Outcomes

The study will examine 2 quantitative and 2 qualitative primary outcomes for the PWD, namely, reduction in behavioral and psychological symptoms in dementia (BPSD), improvement in cognitive function, increase in the feeling of social presence, and an increase in self-awareness and engagement. The primary outcomes for the informal caregiver (family caregivers) are a reduction in carer burden, improvement in carer coping and relief from stress, an increased quality of visits from the informal caregivers to the PWD, and an improvement in the quality of the relationship with their relative with dementia. The primary outcome for the professional caregiver is a reduction in caregiver burnout.

### Secondary Outcomes

Secondary outcomes for the PWD are changes in the prescription of medication, International Classification of Functioning, Disability, and Health (ICF) scores, mortality rate compared with previous existing records, number of hospitalizations comparing control and intervention groups, and an improvement in physical function and balance.

### Exploratory Outcomes

Some interesting outcomes will be measured with an exploratory aim, but because of their nature as explained ahead, they may not allow for clear conclusions to be drawn. Instead, the purpose of these outcomes is to provide preliminary insights that can be built upon in future studies. These outcomes are as follows: a reduction in depressive symptoms and loneliness, relief from feelings related to the care burden, and improvement in quality of life.

The outcomes regarding depression and relief from feelings related to care burden are measured by means of semistructured interviews. The inclusion of people in later stages of dementia means that there may be challenges in applying these measures to all participants in the study.

A total of 4 months is a short amount of time, and therefore, it is not expected that a large improvement in the quality of life will be observed during this period. However, a small change in the quality of life may provide rationale for future longitudinal studies of the use of SENSE-GARDEN in dementia care, in which quality of life can be examined to a greater extent.

Physiological data will also be collected during the SENSE-GARDEN sessions. The Empatica E4 [20] will be used to collect information on electrodermal activity (EDA) and heart rate. These will be assessed during SENSE-GARDEN visits, as reaction to different stimuli. Data will be collected from a subgroup of participants, depending on their tolerance to use a wristband-mounted device. The device is validated for clinical use. Previous research on nonpharmacological interventions has used the E4 wristband to measure engagement and arousal states in people with mild to moderate dementia [21].

## Methods

### User-Centered Design

**Figure 2:**
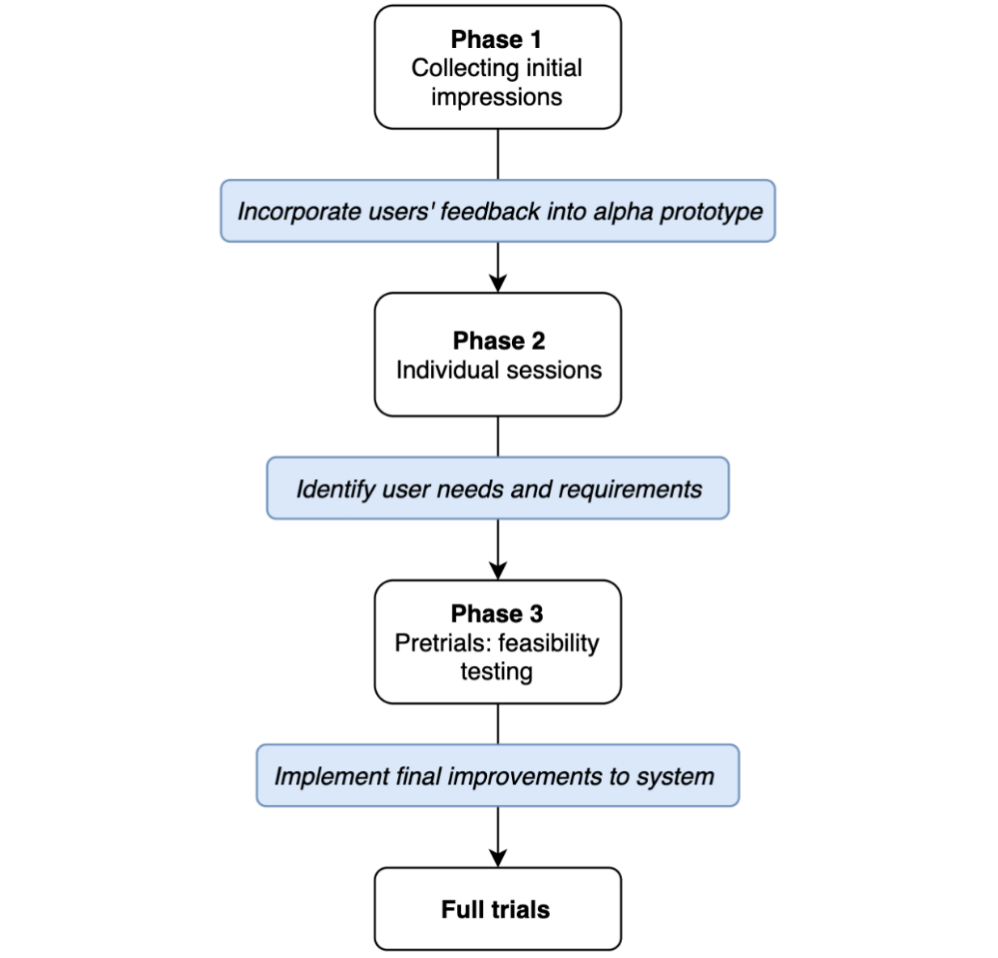
Overview of user centred design process.

To ensure that the SENSE-GARDEN meets the needs of the users, the project has adopted a user-centered design approach to the development of the intervention. This user-centered design process is divided into 3 phases. An overview of the phases is shown in Figure 2.

The first phase focuses on collecting an initial impression of the user experience with nonfunctional low-fidelity prototypes of the SENSE-GARDEN (eg, mock-ups). Small groups of users will be invited to each test site to give their feedback on the overall concept of SENSE-GARDEN. These user groups will include professional care staff, family caregivers, and people living with mild cognitive impairment. Including these individuals at an early stage of the project will ensure that their views are incorporated into the development of SENSE-GARDEN.

The second phase focuses on creating experiences for individual users and aims at gaining a deeper understanding of the users’ needs and requirements. An Alpha version of the SENSE-GARDEN system will be available for this phase of testing. This initial prototype of the SENSE-GARDEN will be tested in a controlled environment. Following the test, a semistructured interview will be conducted with the user to gain a rich insight into their experience.

The third phase involves feasibility testing of the system in the form of pretrials. A small number of users will be recruited at each site (1-2 users). The SENSE-GARDEN test will be tailored to each individual user by using personalized media content, such as family photographs, videos from holidays, and favorite music. The purpose of these tests is to not only ensure that the system works but also define the process of creating individualized experiences for each user.

### Study Design

**Figure 3:**
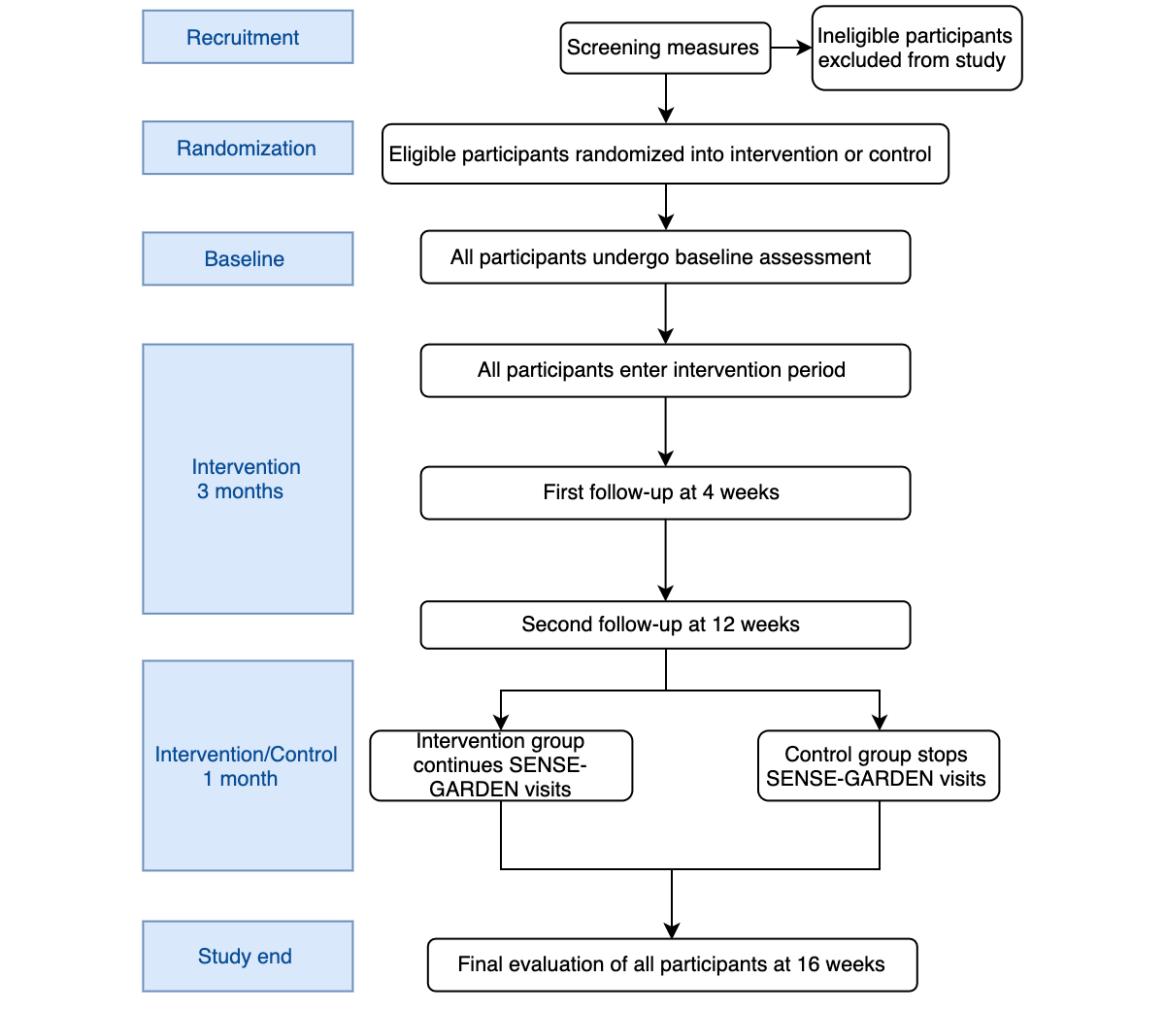
Study timeline.

For the full trials, a controlled before-after study design will be used. The timeline for the study is shown in Figure 3. All participants eligible for the study will receive the SENSE-GARDEN intervention for 3 months. For the final month of the study, half of the participants (the intervention group) will continue to receive the SENSE-GARDEN intervention. The other half of the participants (the control group) will discontinue visits to the SENSE-GARDEN, receiving only normal care. The randomization procedure will use sealed envelopes and will be performed by the local researcher at each test site. The total number of envelopes will be prepared before the recruitment period. A sealed envelope will be assigned to each participant at the time of her or his enrolment. This randomization is to determine whether visits to the SENSE-GARDEN have lasting effects.

The study will start in August 2019, with a recruiting period of 6 months. The intervention period for each participant will be 3 months, followed by 1 month in either the intervention or control condition. The total study period will be 10 months.

### Description of Test Sites

There will be 3 sites in 3 trial countries: Belgium, Norway, and Portugal. With these countries, a large coverage of the European context is achieved, allowing to study cultural, social, economic, and legal differences between the countries and European regions.

The test site in Belgium will be Aan de Beverdijk care home based in Hamont-Achel. This care home is part of the VULPIA group, a care provider that comprises 22 elderly care homes. Aan de Beverdijk focuses on providing tailored care based on the individual needs of its 90 residents. The test site in Norway will be Odda sjukeheim, a municipality-based care home for the elderly based in the center of Hardanger. The test site in Portugal will be the Lar Santa Joana Princesa care home for the elderly. This care home is a part of the Santa Casa de Misericórdia de Lisboa (SCML). SCML operates according to a humanitarian goal, and its care homes focus on promoting the quality of life of its residents.

Although it is inevitable that there will be differences among care homes, the SENSE-GARDEN space at each site is being custom-built for the specific purposes of this project. Each SENSE-GARDEN will have the same equipment, the same software and will be in a closed, private room. Having a controlled space at each test site will help reduce the amount of variability among the 3 countries.

### Participants

This study will recruit a total of 55 PWDs living in care facilities, in a multisite trial with 3 sites. Details for each site are as follows: (1) Belgium—1 study site, 25 PWDs, 25 informal caregivers, and 3 professional caregivers, (2) Norway—1 study site, 15 PWDs, 15 informal caregivers, and 3 professional caregivers, and (3) Portugal—1 study site, 15 PWDs, 15 informal caregivers, and 3 professional caregivers.

### Recruitment

Participants will be recruited at each study site by a professional caregiver from the SENSE-GARDEN team. The caregivers will inform potential participants about the study with the use of an information leaflet about the project. The potential participants who are interested in the study can then voluntarily express their willingness to participate. The participants who do not express an interest will not be contacted by the project team.

Professional caregivers included in the study will have at least 6 months experience in caring for dementia patients and a background in either nursing, occupational therapy, or physiotherapy or another relevant background when supported by long-term professional experience as a caregiver.

Before the study, the professional caregiver will have a 1-week training in SENSE-GARDEN use. This training comprises guidance on how to collect information from the participant and/or their family members for creating a user profile, upload media contents to the SENSE-GARDEN system, create *workflows* of media contents for individual SENSE-GARDEN sessions, and control the numerous elements in the SENSE-GARDEN space.

Each potential participant will undergo an assessment by a health professional for the evaluation of inclusion and exclusion criteria. For the participants fulfilling the criteria, a randomization to the intervention or control group will be performed.

#### Inclusion Criteria

To be included in the study, the participant must be aged ≥55 years and living with dementia in stage 2 (moderate) or stage 3 (severe) according to the Clinical Dementia Rating (CDR) Scale [22], possibly with comorbidities. The participant must also provide informed consent to participate (self-given or given by nominated legal tutor).

#### Exclusion Criteria

Other severe psychiatric disturbance diagnosed by Diagnostic and Statistical Manual of Mental Disorders (4th Edition, Text Revision) criteria, concurrent severe medical condition (extreme or disabling comorbidities), or physical disability not allowing participation in the SENSE-GARDEN activities.

#### Informal Caregivers

The informal caregiver should be a family member or close friend of the PWD. There will be at least 1 informal caregiver for each PWD recruited.

##### Inclusion Criteria

There is no age limit for the informal caregivers; they should have a computer and internet connection and provide signed informed consent to participate.

### Study Procedures

During the 3 months of the intervention period, the PWD will visit the SENSE-GARDEN on an average of 3 times per week. Each participant should visit the SENSE-GARDEN a minimum of 25 times during the intervention period. Any participant who visits less than 25 times will be considered a dropout. On each visit to the SENSE-GARDEN, the PWD is accompanied by 1 caregiver (formal or informal). It is desirable that approximately one-third of the visits are performed with an informal caregiver.

Logs of interaction with the system will be collected continuously, including visit time and duration and system feedback given by the caregivers (through a tablet interface).

Observations will be made at 3 of the SENSE-GARDEN sessions during the intervention period. During this time, measurements on social presence and physical activity will be collected. Video recording will be required during the 3 observation sessions. The recordings will solely be used for data analysis and will not be shared. They will be accessible only to authorized researchers in charge of data analysis. Video recordings will only be made during sessions involving participants that have given their informed consent to being recorded.

For the control period, visits from the family will be logged during normal care.

### Assessment Methods

The methods include the following types of data collection: self-reported measures, questionnaires, interviews, and observations. For interviews and questionnaires requiring the participation of the PWD, the capacity of the PWD to provide information will be considered. In the case of participants that are not competent to provide input, the tests will be performed with a proxy who will provide information on the patient’s behalf. In some cases, the presence of both the PWD and proxy will be considered.

#### Screening Measures

The CDR Scale [22] will be used to assess the severity and progression of the individual’s dementia. Only individuals at level 2 (moderate dementia) or level 3 (severe dementia) will be included in this study.

To decide how to best tailor the SENSE-GARDEN intervention to each individual participant, the Adolescent/Adult Sensory Profile will be used [23]. The Adolescent/Adult Sensory Profile is a tool that evaluates behavioral responses to sensory experiences, with categories focusing on taste and smell, touch, auditory processing, visual processing, movement, and activity level. This measure has recently been tested with people with severe dementia to identify individual sensory processing preferences [24]. This tool will be used in the SENSE-GARDEN study to determine the most appropriate level of sensory stimulation for each participant.

#### Outcome Measures

The following measures will be applied at baseline (T0), 4-week follow-up (T1), 12-week follow-up (T2), and a final follow-up at 16 weeks (T3). An overview of these measures is given in Table 1.

**Table 1:** Overview of outcome measures.

Outcome		Measurement	Timepoint
**Outcome for the person with dementia**			
	A reduction in BPSD^a^	CMAI^b^ and BANS-S^c^	T0^d^, T1^e^, T2^f^, T3^g^
	Improvement in quality of life	QUALID^h^	T0, T1, T2, T3
	Reduction in depressive symptoms and loneliness	CSDD^i^	T0, T1, T2, T3
	Increase in the feeling of social presence	OERS^j^, OME^k^, VNVIS-CR^l^	T1^m^, T2^m^, T3^m^
	Improvement in cognitive function	Mini-Cog^n^, FAST^o^, GDS^p^	T0, T1, T2, T3
	Relief from feelings related to the care burden	Semistructured interview	T3
	Increase in self-awareness and engagement	Audio recordings analyzed using conversation analysis	T1^m^, T2^m^, T3^m^
	ICF^q^ scores	WHODAS 2.0^r^	T0, T1, T2, T3
	Prescription of medication	Medical records	T0, T1, T2, T3
	Mortality rate compared with previous existing records	Medical records	T0, T1, T2, T3
	Number of hospitalizations comparing control and intervention groups	Medical records	T0, T1, T2, T3
	Exploration of physiological data including electrodermal activity and heart rate	Empatica E4 wristband	T1^m^, T2^m^, T3^m^
	Improvement in physical function and balance	FRT^s^	T0, T1, T2, T3
**Outcome for the informal caregiver**			
	A reduction in caregiver burden	ZBI^t^	T0, T1, T2, T3
	Improvement in caregiver coping and relief from stress	Brief-COPE^u^	T0, T1, T2, T3
	An increased quality of visits to the person with dementia	FAVS-D^v^	T0, T1, T2, T3
	An improvement in the quality of relationship with the person with dementia	QCPR^w^	T0, T1, T2, T3
**Outcome for the formal caregiver**			
	Reduction in caregiver burnout	MBI-HSS^x^	T0, T1, T2, T3

^a^BPSD: behavioral and psychological symptoms of dementia.

^b^CMAI: Cohen–Mansfield Agitation Inventory.

^c^BANS-S: Bedford Alzheimer Nursing Scale–Severity.

^d^T0: baseline.

^e^T1: 4-week follow-up.

^f^T2: 12-week follow-up.

^g^T3: 16-week follow-up.

^h^QUALID: Quality of Life in Late Stage Dementia scale.

^i^CSDD: Cornell Scale for Depression in Dementia.

^j^OERS: Observed Emotion Rating Scale.

^k^OME: Observational Measurement of Engagement.

^l^VNVIS-CR: Verbal and Nonverbal Interaction Scale.

^m^Measurement to be taken during SENSE-GARDEN session.

^n^Mini-Cog: 3-min instrument to screen for cognitive impairment in older adults.

^o^FAST: Functional Assessment Staging Tool.

^p^GDS: Global Deterioration Scale.

^q^ICF: International Classification of Functioning, Disability and Health.

^r^WHODAS 2.0: World Health organization Disability Assessment Schedule 2.0.

^s^FRT: Functional Reach Test.

^t^ZBI: Zarit Burden Interview.

^u^Brief-COPE: abbreviated version of the Coping Orientation to Problems Experienced inventory, a self-report questionnaire.

^v^FAVS-D: Family Visit Scale for Dementia.

^w^QCPR: Quality of Carer Patient Relationship scale.

^x^MBI-HSS: Maslach Burnout Inventory–Human Services Survey.

##### Person With Dementia

###### Primary Outcomes

####### A Reduction in Behavioral and Psychological Symptoms in Dementia

The Bedford Alzheimer Nursing Scale–Severity (BANS-S) [25] and the Cohen–Mansfield Agitation Inventory (CMAI) [26] will be used to determine whether the intervention reduces behavioral and psychological symptoms of dementia. BANS-S is a nursing staff–administered questionnaire that assesses dressing, sleeping, speech, eating, mobility, muscles, and eye contacts in persons with severe dementia. It is a reliable measure with good internal consistency (Cronbach alpha=.64-.80). CMAI is a widely used tool that evaluates aggressive behavior, nonaggressive behavior, and verbally aggressive behavior. The caregiver-rated questionnaire comprises 29 agitated behaviors, each rated on a 7-point scale of frequency ranging from 1 (never) to 7 (several times an hour).

####### Increase in the Feeling of Social Presence

Observational measures will be used to determine the level of social engagement in the participants with dementia. These tools will be the Observed Emotion Rating Scale (OERS) [27], the Verbal and Nonverbal Interaction Scale (VNVIS-CR) [28], and the Observational Measurement of Engagement (OME) [29]. All 3 measures have previously been used in studies observing people living with dementia. OERS assesses pleasure, general alertness, anxiety or fear, and sadness at 10-min intervals. VNVIS-CR is a tool developed specifically to measure verbal and nonverbal interaction in people with mild to moderate dementia. The 26 items assess nonverbal social behaviors, nonverbal unsociable behaviors, verbal sociable behaviors, and verbal nonsociable behaviors. The OME evaluates various dimensions of engagement in people with mild to severe dementia. The tool measures attention to stimulus, attitude toward stimulus, rate of refusal, duration, and activity.

Observations will be made during at least 2 of the SENSE-GARDEN sessions for each participant. For participants in the intervention group, recordings will be taken during a SENSE-GARDEN session in week 4, week 12, and week 16. Participants randomized to the control group will only have recordings taken during a session in week 4 and week 12, as these individuals will no longer be visiting the SENSE-GARDEN after week 12.

####### Improvement in Cognitive Function

The Mini-Cog [30], the Functional Assessment Staging Tool (FAST) [31], and the Global Deterioration Scale (GDS) [32] will be used to assess whether there is any improvement in cognitive function among the participants. The Mini-Cog is an assessment of cognitive function that only takes 2 to 5 min to complete. It comprises a word recall task and a clock drawing test. FAST is a dementia staging tool that focuses on an individual’s level of functioning and ability to carry out activities of daily living. Scores range from 0 to 7, with a higher score indicating an increased level of functional decline. GDS is a brief dementia staging scale that assesses the stage and progression of dementia in terms of cognitive decline. Scores range from 0 to 7, with a higher score indicating a more severe level of cognitive decline.

####### Increase in Self-Awareness and Engagement

Conversation analysis will be used to assess the PWD’s level of self-awareness and engagement with both the SENSE-GARDEN stimuli and the caregiver conducting the session. To conduct conversation analysis, audio recordings of certain SENSE-GARDEN sessions will be taken.

###### Secondary Outcomes

Medical records will be used to assess prescription of medication and mortality rate compared with previous existing record, and they will also be used to compare the number of hospitalizations between the control and intervention groups.

ICF scores will be collected using the WHO Disability Assessment Schedule 2.0 (WHODAS 2.0) [33]. The WHODAS 2.0 is a tool for measuring functioning and disability in accordance with the ICF framework. The tool assesses 6 domains of functioning: cognition, mobility, self-care, getting along with others, life activities, and participation in community activities. This study will use the 36-item version that can either be self-administered or administered by a proxy or interviewer.

The Functional Reach Test (FRT) [34] will be used to assess any improvement in physical function and balance.

The FRT is a widely used tool to screen for balance problems in older adults. The test comprises a brief physical task that asks the individual to reach forward without moving his or her feet. The test measures the amount of maximum excursion that the individual is able to cover without losing balance or taking a step.

###### Exploratory Outcomes

####### Physiological Data

The Empatica E4 [20] will be used to measure physiological responses to the SENSE-GARDEN stimuli. These physiological responses will include heart rate and EDA.

####### A Reduction in Depressive Symptoms and Feelings of Loneliness

The Cornell Scale for Depression in Dementia (CSDD) [35] will be used to determine any reduction in depressive symptoms and feelings of loneliness in PWDs. The CSDD is a measure of depression among people with moderate to severe dementia, making it an appropriate choice for the SENSE-GARDEN study. The 19-item tool assesses mood-related signs of depression, behavioral disturbance, physical signs, cyclic functions, and ideational disturbance. It is administered in the form of a semistructured interview by a health care professional or clinician.

####### An Improvement in Quality of Life

The Quality of Life in Late Stage Dementia (QUALID) scale [36] is the only scale developed for advanced dementia and will, therefore, be used to determine any improvement in quality of life in this study. It was created to appreciate the outcome of clinical management, including the effect of therapeutic interventions. QUALID contains 11 items that describe observable behaviors, such as emotional state, interaction with others, aggression, and physical signs of discomfort. It is completed by a health care professional or a family caregiver.

####### Relief From Feelings Related to the Care Burden

A semistructured dyadic interview will be conducted with the PWD and their family caregiver at the end of the study period. This interview will be used to explore the relationship between the 2 individuals. One factor will be the relief from feelings related to the care burden.

###### Informal (Family) Caregiver

####### A Reduction in Caregiver Burden

The Zarit Burden Interview [37] will be used to measure caregiver burden. The tool is a self-report measure that evaluates a caregiver’s health, psychological well-being, finances, social life, and relationship shared with the PWD. Scores range from 0 to 88, with a higher score indicating a higher level of burden.

####### An Improvement in Caregiver Coping and Relief From Stress

The Brief-COPE [38] is a shortened version of the Coping Orientation to Problems Experienced (COPE) inventory [39] and will be used to determine any improvement in the coping strategies in family caregivers. The Brief-COPE is a self-reported measure comprising 28 items that assess the following coping strategies in caregivers: emotion-focused strategies, problem-focused strategies, and dysfunctional coping strategies.

####### An Increased Quality of Visits to the Person With Dementia

The Family Visit Scale for Dementia [40] will be used to evaluate the quality of visits from the family members to the participants with dementia. The questionnaire is completed by the family member and evaluates nursing staff interaction with residents and visitors, meaningfulness of the visit, cleanliness, and connection established between the visitor and the resident.

####### An Improvement in the Quality of the Relationship With the Person With Dementia

The Quality of Carer Patient Relationship scale [41] will be used to assess an improvement in the relationship between the PWD and the family caregiver. This scale comprises 14 items that assess the level of warmth in the relationship and the absence of criticism.

###### Formal (Professional) Caregiver

####### A Reduction in Caregiver Burnout

The Maslach Burnout Inventory–Human Services Survey (MBI-HSS) [42] will be used to assess caregiver burnout in professional caregivers. The MBI-HSS is an extensively used tool for measuring burnout in professionals working in human services. The tool focuses on emotional exhaustion, depersonalization, and personal accomplishment.

### Data Analysis

Quantitative data will be analyzed using SPSS Statistics version 25 (IMB Corp). Stratification by gender, age, and type and stage of dementia will be used to analyze the data. Repeated measures analysis of variance (ANOVA) will be used to assess whether the SENSE-GARDEN intervention has an effect on participants over time. Given the novelty of the SENSE-GARDEN intervention, it is not possible to assess the previous literature for clinically meaningful differences. However, Cohen *d* has been used to determine effect size in studies focusing on the effects of nonpharmacological interventions on behavioral outcomes in PWDs [43].

Cohen *d* is a measure of standardized difference between 2 means [44]. It uses standard deviation units to express the magnitude of difference between the 2 means, indicating the importance of the difference. Cohen has defined a small effect size as *d*=0.2, a medium effect size as *d*=0.5, and a large effect size as *d*=0.8.

For this study, repeated measures ANOVA will be performed on the data using SPSS to assess the effects of the intervention over the numerous time points (T0-T3). We estimate that a sample size of 55 will have over 80% power to detect a medium difference between means at a .05 significance level.

Qualitative interview data will be analyzed using thematic analysis [45]. Thematic analysis is a method of identifying patterns of prevalent ideas or responses and can offer rich insight into the attitudes and beliefs of participants. This aspect of the study will be vital for understanding the users’ experiences of the SENSE-GARDEN.

### Ethical Approval and Considerations

Applications for ethical approval have been submitted by the 3 study sites in accordance to the national regulation. This study has been submitted to Norway’s Regional Committee for Medical and Health Research Ethics and is currently under assessment (document ID 1094463).

#### Confidentiality and Privacy

Confidentiality of private health information will be ensured according to the regulation (EU) 2016/679 (General Data Protection Regulation). All private personal data will be deidentified: every unique identifying number, characteristic, or code identifier of the individual, relatives, or employers will be removed, so that the information can be used alone or in combination with other information. The resulting data will be analyzed by a statistician to ensure that no individually identifiable health information remains.

#### Informed Consent

The consent of participants will be required. They will be provided a letter of informed consent to be signed, together with an information sheet. The participants will be informed regarding the study’s aims and protocol and what involvement the study will comprise. The participants will be made aware of their right to withdraw from the study at any time. Information regarding confidentiality and data protection will be given. The letter of consent will be signed by the patient, when competent, or otherwise a legal tutor, signing as proxy.

#### Exit Strategy

An important ethical consideration for users is the exit strategy at the end of the project. All care organizations in the consortium have initially expressed their interest in evaluating the potential of keeping the SENSE-GARDEN after project end. The YOUSE GmbH method will be used also in connection with the exit strategy.

## Results

The trials commenced recruitment in August 2019. All data are expected to be collected by the end of May 2020. All phases of the user-centered design process have taken place, which has helped shape the development of SENSE-GARDEN. The results of the first phase have been published and presented at the Fourth International Conference on Human and Social Analytics 2018 (in press). Interviews with 52 users comprising people with mild cognitive impairment and informal and formal caregivers were conducted in November 2017. The aims of these interviews were to collect initial responses and attitudes toward the SENSE-GARDEN concept and investigate what benefits, if any, the users thought SENSE-GARDEN could provide in the care of PWDs.

The interviews were analyzed using thematic analysis [45]. A total of 6 themes were identified: benefits for all, shared experiences, past and present, focus on the individual, emotional stimulation, and challenges to consider. The ideas expressed by the users provided rich insights into how the SENSE-GARDEN intervention should be implemented. For example, a point raised by the users was the importance of caregiver facilitation. Although it is important for the SENSE-GARDEN system to work correctly, it will be essential to involve a caregiver who is able to facilitate the session in a safe and effective way. From this feedback, we are working on creating user training materials that will help the caregivers use the system and also aid them in creating meaningful experiences for the PWD.

Results from the second phase of the user-centered design process emphasized the importance of including personalized media contents. At the Norwegian test site, 3 user tests were conducted in a room with a prototype of the SENSE-GARDEN. The first test involved an 85-year-old lady with early-stage dementia and a professional caregiver. This lady did not feel *connected* to the media and also felt that the session was too fast. The second test involved an 89-year-old lady with early-stage dementia and a family caregiver. This session was extremely positive, with the user expressing positive emotions toward the films played during the session. The user was only able to remember watching 1 of the videos played during the session (a video of Norwegian folk dancing); however, she spoke about this video fondly during the semistructured interview after the session. Finally, the third test involved an older adult aged 85 years without cognitive impairment and 2 professional caregivers. This was again a very positive session, and the 3 individuals enjoyed talking together about the videos that they were watching. Testing at the other sites has also affirmed that users respond better to personalized or familiar media contents.

**Figure 4:**
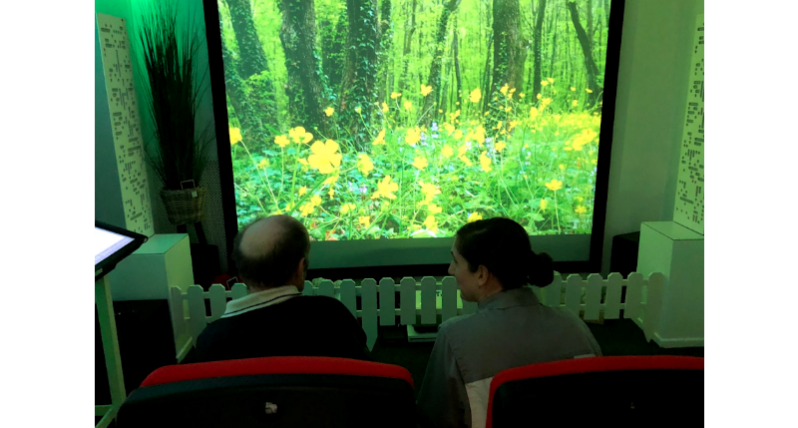
Photo of a test session using a prototype of SENSE-GARDEN.

The third phase of user testing has been completed, which evaluated functional prototypes of the SENSE-GARDEN system. An example of a SENSE-GARDEN prototype is shown in Figure 4. Results from this feasibility testing are expected to be submitted for publishing in October 2019.

## Discussion

### Summary

This paper has outlined the objectives, study design, and preliminary results of an AAL-funded study, SENSE-GARDEN. To our knowledge, SENSE-GARDEN is the first intervention to combine multisensory stimuli, virtual technology, and autobiographical material to create an immersive and personalized room for PWDs. Our progress thus far has indicated the value of involving user groups in the initial stages of intervention development and has shown that these users have a positive outlook toward SENSE-GARDEN. The evaluation summary report has been provided in Multimedia Appendix 1.

### Limitations

A limitation of this study proposal is its small sample size. However, although the study may not be able to produce generalizable results, it will provide important insights into the use of a novel technology, such as SENSE-GARDEN for PWDs. The results will provide foundation for further study in the use of immersive, individualized environments and how these environments can be used to facilitate communication and person-centered care in residential care home environments.

A further limitation is the potential variance between the test sites. Implementing this study protocol in 3 different countries may be difficult in terms of ensuring that the SENSE-GARDEN intervention is delivered in the same manner in each care home. Cultural differences between the test sites may mean that methods of facilitation from care home staff could affect the overall SENSE-GARDEN experience, therefore, influencing the results from the study. However, all professional care staff will be given the same training on how to use the SENSE-GARDEN. This training should help to ensure that the intervention is conducted in a similar way at each test site.

### Conclusions

Despite these limitations, this study has the potential to provide important contributions to current research on dementia care. The interdisciplinary nature of this project will allow us to evaluate the SENSE-GARDEN from multiple perspectives, such as technical, sociological, and psychological. The findings from the full trials have the potential to offer numerous implications on future research in dementia care and also in promoting person-centered care in practice.

## Appendix

Multimedia Appendix 1 Active Assisted Living evaluation summary report.

## Abbreviations

AAL: Active Assisted Living
ANOVA: analysis of variance
BANS-S: Bedford Alzheimer Nursing Scale–Severity
BPSD: behavioral and psychological symptoms of dementia
CDR: Clinical Dementia Rating
CMAI: Cohen–Mansfield Agitation Inventory
CSDD: Cornell Scale for Depression in Dementia
EDA: electrodermal activity
FAST: Functional Assessment Staging Tool
FRT: Functional Reach Test
GDS: Global Deterioration Scale
ICF: International Classification of Functioning, Disability and Health
MBI-HSS: Maslach Burnout Inventory–Human Services Survey
OERS: Observed Emotion Rating Scale
OME: Observational Measurement of Engagement
PWDs: people with dementia
QUALID: Quality of Life in Late Stage Dementia scale
SCML: Santa Casa de Misericórdia de Lisboa
VNVIS-CR: Verbal and Nonverbal Interaction Scale
WHO: World Health Organization
WHODAS 2.0: WHO Disability Assessment Schedule 2.0

## References

[1] (2017). Global Action Plan on the Public Health Response to Dementia 2017 - 2025.

[2] Prince M, Wimo A, Guerchet M, Ali GC, Wu YT, Prina M (2015). Alzheimer's Disease International.

[3] Savundranayagam MY, Hummert ML, Montgomery RJ (2005). Investigating the effects of communication problems on caregiver burden. J Gerontol B Psychol Sci Soc Sci.

[4] Kovach CR, Noonan PE, Schlidt AM, Wells T (2005). A model of consequences of need-driven, dementia-compromised behavior. J Nurs Scholarsh.

[5] Burgener S, Twigg P (2002). Relationships among caregiver factors and quality of life in care recipients with irreversible dementia. Alzheimer Dis Assoc Disord.

[6] Smit D, de Lange J, Willemse B, Twisk J, Pot AM (2016). Activity involvement and quality of life of people at different stages of dementia in long term care facilities. Aging Ment Health.

[7] Hancock GA, Woods B, Challis D, Orrell M (2006). The needs of older people with dementia in residential care. Int J Geriatr Psychiatry.

[8] Cadieux MA, Garcia LJ, Patrick J (2013). Needs of people with dementia in long-term care: a systematic review. Am J Alzheimers Dis Other Demen.

[9] Milte R, Shulver W, Killington M, Bradley C, Ratcliffe J, Crotty M (2016). Quality in residential care from the perspective of people living with dementia: the importance of personhood. Arch Gerontol Geriatr.

[10] Lee KH, Algase DL, McConnell ES (2013). Daytime observed emotional expressions of people with dementia. Nurs Res.

[11] Finnema E, Dröes RM, Ribbe M, van Tilburg W (2000). The effects of emotion-oriented approaches in the care for persons suffering from dementia: a review of the literature. Int J Geriatr Psychiatry.

[12] Gowans G, Campbell J, Alm N, Dye R, Astell A, Ellis M (2004). Designing a Multimedia Conversation Aid for Reminiscence Therapy in Dementia Care Environments. CHI'04 Extended Abstracts on Human Factors in Computing Systems.

[13] Moyle W, Jones C, Dwan T, Petrovich T (2018). Effectiveness of a virtual reality forest on people with dementia: a mixed methods pilot study. Gerontologist.

[14] Cui Y, Shen M, Ma Y, Wen SW (2017). Senses make sense: an individualized multisensory stimulation for dementia. Med Hypotheses.

[15] Critten V, Kucirkova N (2019). 'It brings it all back, all those good times; it makes me go close to tears'. Creating digital personalised stories with people who have dementia. Dementia (London).

[16] Damianakis T, Crete-Nishihata M, Smith KL, Baecker RM, Marziali E (2010). The psychosocial impacts of multimedia biographies on persons with cognitive impairments. Gerontologist.

[17] Subramaniam P, Woods B (2016). Digital life storybooks for people with dementia living in care homes: an evaluation. Clin Interv Aging.

[18] Purves B, Savundranayagam MY, Kelson E, Astell AJ, Phinney A, Resnick B, Gwyther LP, Roberto KA (2011). Fostering resilience in dementia through narratives: contributions of multimedia technologies. Resilience in Aging: Concepts, Research, and Outcomes.

[19] (1995). Constitution of the World Health Organization: Basic Documents.

[20] (2019). Empatica: Embrace Watch | Smarter Epilepsy Management.

[21] Perugia G, Rodríguez-Martín D, Boladeras MD, Mallofré AC, Barakova E, Rauterberg M (2017). Electrodermal Activity: Explorations in the Psychophysiology of Engagement With Social Robots in Dementia. Proceedings of the 26th IEEE International Symposium on Robot and Human Interactive Communication.

[22] Hughes CP, Berg L, Danziger WL, Coben LA, Martin RL (1982). A new clinical scale for the staging of dementia. Br J Psychiatry.

[23] Brown C, Dunn W (2002). Adolescent-Adult Sensory Profile: User's manual.

[24] Ravn MB, Klingberg T, Petersen KS (2018). The adult sensory profile™ in care homes targeting people diagnosed with dementia: a qualitative study from the care provider perspective. Rehabil Res Pract.

[25] Volicer L, Hurley AC, Lathi DC, Kowall NW (1994). Measurement of severity in advanced Alzheimer's disease. J Gerontol.

[26] Cohen-Mansfield J, Marx MS, Rosenthal AS (1989). A description of agitation in a nursing home. J Gerontol.

[27] Lawton MP, van Haitsma K, Klapper J (1996). Observed affect in nursing home residents with Alzheimer's disease. J Gerontol B Psychol Sci Soc Sci.

[28] Williams CL, Newman D, Hammar LM (2017). Preliminary psychometric properties of the verbal and nonverbal interaction scale: an observational measure for communication in persons with dementia. Issues Ment Health Nurs.

[29] Cohen-Mansfield J, Dakheel-Ali M, Marx MS (2009). Engagement in persons with dementia: the concept and its measurement. Am J Geriatr Psychiatry.

[30] Borson S, Scanlan J, Brush M, Vitaliano P, Dokmak A (2000). The mini-cog: a cognitive 'vital signs' measure for dementia screening in multi-lingual elderly. Int J Geriatr Psychiatry.

[31] Reisberg B (1988). Functional assessment staging (FAST). Psychopharmacol Bull.

[32] Reisberg B, Ferris SH, de Leon MJ, Crook T (1982). The Global Deterioration Scale for assessment of primary degenerative dementia. Am J Psychiatry.

[33] Ustün TB, Chatterji S, Kostanjsek N, Rehm J, Kennedy C, Epping-Jordan J, Saxena S, von Korff M, Pull C, WHO/NIH Joint Project (2010). Developing the World Health Organization disability assessment schedule 2.0. Bull World Health Organ.

[34] Duncan PW, Weiner DK, Chandler J, Studenski S (1990). Functional reach: a new clinical measure of balance. J Gerontol.

[35] Alexopoulos GS, Abrams RC, Young RC, Shamoian CA (1988). Cornell scale for depression in dementia. Biol Psychiatry.

[36] Weiner MF, Martin-Cook K, Svetlik DA, Saine K, Foster B, Fontaine CS (2000). The quality of life in late-stage dementia (QUALID) scale. J Am Med Dir Assoc.

[37] Zarit SH, Reever KE, Bach-Peterson J (1980). Relatives of the impaired elderly: correlates of feelings of burden. Gerontologist.

[38] Carver CS (1997). You want to measure coping but your protocol's too long: consider the brief COPE. Int J Behav Med.

[39] Carver CS, Scheier MF, Weintraub JK (1989). Assessing coping strategies: a theoretically based approach. J Pers Soc Psychol.

[40] Volicer L, DeRuvo L, Hyer K, Piechniczek-Buczek J, Riordan ME (2008). Development of a scale to measure quality of visits with relatives with dementia. J Am Med Dir Assoc.

[41] Spruytte N, van Audenhove C, Lammertyn F, Storms G (2002). The quality of the caregiving relationship in informal care for older adults with dementia and chronic psychiatric patients. Psychol Psychother.

[42] Maslach C, Jackson SE, Leiter MP, Schaufeli WB, Schwab RL (1986). Maslach Burnout Inventory Manual.

[43] Low LF, Brodaty H, Goodenough B, Spitzer P, Bell J, Fleming R, Casey A, Liu Z, Chenoweth L (2013). The Sydney multisite intervention of LaughterBosses and ElderClowns (SMILE) study: cluster randomised trial of humour therapy in nursing homes. BMJ Open.

[44] Cohen J (1988). Statistical Power Analysis for the Behavioral Sciences. Second Edition.

[45] Braun V, Clarke V (2006). Using thematic analysis in psychology. Qual Res Psychol.

[46] Goodall G, Ciobanu I, Broekx R, Sørgaard J, Anghelache I, Anghelache-Tutulan C, Diaconu M, Mæland S, Borve T, Dagestad A, Bormans P, Custers M, Losleben K, Valadas R, de Almeida CV, Matias A, Marin A, Taraldsen K, Maetzler W, Berteanu M, Serrano JA (2019). The role of adaptive immersive technology in creating personalised environments for emotional connection and preservation of identity in dementia care. Int J Adv Life Sci.

