# Supplementary material for: The Use of Virtual and Immersive Technology in Creating Personalized Multisensory Spaces for People Living With Dementia (SENSE-GARDEN): Protocol for a Multisite Before-After Trial
Source: JMIR Res Protoc. 2019 Aug 19;8(9):e14096. doi: 10.2196/14096 (PMC6786690; doi:10.2196/14096)
Supplement: Multimedia Appendix 1 [file resprot_v8i9e14096_app1.pdf]

The Active Assisted Living Programme

Evaluation Summary Report (ESR) -

Evaluation-call-2016

PROPOSAL: SENSE-GARDEN

AAL-CALL-2016-054

1. RELEVANCE AND SCOPE	4 / PASSED

The SENSE GARDEN proposal aims to create personalised garden like environments as a therapeutic solution for people with dementia. The proposed system will let them reminisce in immersive and stimulating environments.
The proposed solution addresses well the Call challenges. It provides an interesting ICT solution to enhance the wellbeing of dementia patients. The innovation will be a technology-rich room equipped with multimedia, scents release, and with personal archives for each user. The concept is scientifically and technically sound, though there is almost no new technology as the consortium will integrate existing solutions.

As the proponents plan to install exercise stationary bikes and balance trainers, there will also be the possibility to promote physical activity. The scope is well focused, the objectives are clear.
At proposal stage the consortium has not adequately outlined and discussed how the patients will accept leaving the space, or not being able to freely access it, facing the physical limitations of the area, being disappointed with the artificiality of the artefacts. These aspects will be studied during the project and the net outcome will be positive i.e. the innovation will offer new ways to engage patients.

The example scenario is well described and achievable. The quantitative and qualitative success parameters of the proposal address well the main aspects of the project as criteria to assess the end-users' actual involvement, number of installations and tests, etc… These are appropriate and credible. Criteria to assess the user's satisfaction, quality of life and business success are reported but their relation to accurate quantitative parameters was not adequately provided.

2. QUALITY OF PROPOSED SOLUTION AND THE WORKPLAN	4 / PASSED

The innovation is intended for people in intermediate stages of dementia or with advanced dementia. Also, such populations will be studied within the project. This is a new concept in this environment and it outlines a clear case to demonstrate why this proposal is an advance of the state of the art. The ICT aims at creating a friendly and therapeutic environment away from home. The description of the ICT is clear and convincing.

A comparison of the proposed solution vs. managed care facilities situated in beautiful or at-

tractive locations like in forests, over the sea, in mountains etc. was not properly addressed. The proposal does not adequately outline and discuss if the proposed artificial space is effectively able to compete with those "natural" solutions.

The proposal has credibility concerning the expertise, validation methodologies and assessment instructions.
The users will be involved in all important steps through trials/research groups. Continuous and incremental development of features is planned and the project allows for the collection of long-term experience. The consortium properly outlines how they will obtain participation of PwD and sets out related inclusion criteria. The groups chosen match closely the target market profile. Effective involvement of users in business modelling was not likewise adequately outlined and documented.

The proposal sets out a realistic plan for prototyping and testing the systems. The availability of functional prototypes that users can start testing is predicted already in year one of the project and a working prototype at the end of the project.
The exit strategy is well thought and appropriate with a sound method and concrete roadmap described both in the proposal description and in the work to be performed (Task 2.7).

The work plan is well designed, the work packages are well structured and coherent given the realistic timeframe and tasks duration, the iterative approach adopted to effectively exploit the users' feedback and the compliance with the requirement of starting trials after 1 year since the start of the project. The deliverables and milestones are adequately related to and coherent with the project objectives.

3. QUALITY OF THE CONSORTIUM AND PROJECT MANAGEMENT	4 / PASSED

The consortium has considerable experience of the AAL programme and the consortium leader (UNN) has been a previous winner of the AAL Awards. This gives confidence in the quality and the composition of a well-balanced team.
The consortium has also provided substantial evidence that it has the required expertise and resources to support successful end-user participation.

The governance strategy is appropriate for this type of project. Risk and contingency management is well structured but a comprehensive initial risk assessment of the project was not properly outlined to effectively demonstrate that all aspects of the project have been considered. For instance, at proposal stage, real life experiences, abuse, technical malfunctioning and other issues were not properly described and discussed.

Ethical, regulatory, and privacy issues are taken seriously. Sound and proper techniques to manage personalised data will be adopted providing an adequate level of protection of sensitive data related to personal life history.

The resources are partially in proportion with the scale and complexity of the work described. The resources that appear inadequate for this purpose include those for the production of private media content items. This assessment is based on the fact that the Consortium did not demonstrate having effectively considered the actual effort which this process requires.

The participation of industry partners is adequate.

The IPR strategy is realistic and aligns with the project's expectations. Background and foreground knowledge management is appropriately addressed, with open source philosophy, a partner in the consortium with relevant responsibility and a dedicated task.

4. POTENTIAL IMPACT OF THE PROPOSED SOLUTION ON QUALITY OF LIFE	4 / PASSED

The project's concept consists of immersive and customized spaces developed with proper technologies which may eventually grow beyond the project, thanks to open source based solutions and the use of standard means of communication. The likelihood of a positive impact on the quality of life of people with dementia is high.
There are risks and potential unexpected effects, but the proposal is convincing that for the majority of users the product will be beneficial.

Conversely, social benefits were not likewise treated well. The adoption of the proposed solution will increase the costs for care organizations and the Consortium did not effectively clarify how and if this aspect might be sustainable especially for public care organizations.

The project outcomes are focused on the development of a sense-garden. These are adequately adaptable to changing end users' wishes, needs and abilities. In particular, the solution may be developed to support other disabled populations.

**5. POTENTIAL IMPACT OF THE PROPOSED SOLUTION ON MARKET DEVELOPMENT**	**3 / PASSED**

The product has global potential. The international commercialization will be facilitated by SMEs in all 5 countries in the consortium, covering the West, South, North and East of Europe, thus also providing access to multicultural market feedback. The Consortium used the business model canvas to assess the targeted market. This gives a good framework for exploring the market opportunity and the creation of an initial business model for the project.
Business planning is adequate as it mainly focused on the identification of customer segments, value proposition, channels and customer relationships. Further important parts of the adopted Business Model Canvas template "were defined but left out of this presentation because of template space limitations" (by quoting the project team itself). Anyway, resources allocation is good and three SMEs in the Consortium will contribute to commercialize the product.

The outlined marketing strategy and the envisaged costly business model (cost of delivery vs price point) are the main weaknesses: the pricing policy was not adequately outlined and discussed to demonstrate the effective sustainability of the proposed solution.

The proposals for developing promotional activities demonstrate an initial understanding of the relevant messages and audience groups. This approach is partially adequate because main conferences, journals to target technical and scientific events were outlined and reported; but the preparation and delivery of a proper dissemination plan is missing in the work plan.

**REMARKS**	**NOT RATED**

It is worth pointing out that in the table "Total costs and total breakdown" on page 25, CPX partner will participate with two different teams: one based in Romania and the other based in The Netherlands.
This aspect is not reflected in Part A of the proposal.

TOTAL SCORE: 19
